# Artificial light at night causes an unexpected increase in oxalate in developing male songbirds

**DOI:** 10.1093/conphys/coy005

**Published:** 2018-02-16

**Authors:** Thomas Raap, Rianne Pinxten, Marcel Eens

**Affiliations:** 1 Department of Biology, Behavioural Ecology and Ecophysiology Group, University of Antwerp, Wilrijk, Belgium; 2 Faculty of Social Sciences, Antwerp School of Education, University of Antwerp, Antwerp, Belgium

**Keywords:** Artificial light at night, development, light pollution, oxalate, oxalic acid, sleep

## Abstract

Artificial light at night (ALAN) is a widespread and increasing environmental pollutant with known negative impacts on animal physiology and development. Physiological effects could occur through sleep disruption and deprivation, but this is difficult to quantify, especially in small developing birds. Sleep loss can potentially be quantified by using oxalate, a biomarker for sleep debt in adult humans and rats. We examined the effect of ALAN on oxalate in free-living developing great tits (*Parus major*) as effects during early-life could have long-lasting and irreversible consequences. Nestlings’ physiology was quantified at baseline (= 13 days after hatching) and again after two nights of continued darkness (control) or exposure to ALAN (treatment). We found that ALAN increased oxalate levels but only in male nestlings, rather than decreasing it as was found in sleep-deprived humans and rats. Our results using developing birds differ strongly from those obtained with adult mammals. However, we used ALAN to reduce sleep while in rats forced movement was used. Finally, we used free-living opposed to laboratory animals. Whether oxalate is a reliable marker of sleep loss in developing great tits remains to be examined. Potentially the increase of oxalate in male nestlings was unrelated to sleep debt. Nonetheless, our results substantiate physiological effects of ALAN in developing animals and may provide a foundation for future work with free-living animals.

## Teaser

We quantified the effect of artificial light at night on oxalate, a potential marker of sleep debt, in free-living developing songbirds. Rather than a decrease, as was found in sleep-deprived mammals, oxalate increased in male nestlings. This research indicates a sex-specific physiological impact of ALAN that warrants further investigation.

## Introduction

Artificial light at night (ALAN), also known as light pollution, has greatly altered the night-time environment. It is a widespread, ever increasing and important anthropogenic environmental pressure on wildlife (Falchi *et al.*, 2016; Hölker *et al.*, 2010). ALAN affects a wide variety of behavioural traits, such as reproduction, foraging and migration and has clear deteriorating physiological effects such as a supressed immune response to challenges and increased corticosterone levels (see e.g. Bedrosian *et al.*, 2011; Russ *et al.*, 2015, 2017; reviewed in Russart and Nelson, 2017). Disruption of the immature circadian system during development by ALAN may, due to its effects on the developing brain, influence adult behaviour, physiology, health, and disease and could have long-lasting and irreversible consequences (Fonken and Nelson, 2016). In wild developing great tits (*Parus major*) ALAN has been shown to affect physiology (Raap *et al.*, 2016a, 2016b) and to cause begging at night (Raap *et al.*, 2016c). Sleep disruption due to ALAN, similar as has been demonstrated in adult great tits (Raap *et al.*, 2015, 2016c), seems likely but is difficult to quantify in nestlings. During the nestling period ALAN reduces female great tit sleep behaviour by ≈50% with some females not sleeping at all during the entire night. Furthermore, when nestlings were in natural darkness they never begged for food, while they showed substantial begging behaviour throughout the night when being exposed to ALAN (Raap *et al.*, 2016c). This together with the severe sleep disruption in females gives a strong indication of sleep disruption and loss in great tit nestlings, but this is difficult to quantify as they do not exhibit sleep behaviour like adults. In adults, but not in nestlings, sleep behaviour can be defined as when an individual is in the classical sleep position, with the beak pointing backwards and tucked under the scapulars (Amlaner and Ball, 1983). Sleep is an important behaviour widespread across the animal kingdom (Cirelli and Tononi, 2008; Siegel, 2008) but difficult to study in the field (Rattenborg *et al.*, 2017), especially in small animals.

Recently, a laboratory study showed that sleep loss reduced plasma levels of oxalic acid, also known as oxalate, in humans and in rats (Weljie *et al.*, 2015). Ouyang *et al.* (2017) claimed that light pollution decreased oxalate levels in adult great tits. However, because of several severe methodological and statistical issues with this study, such as a high uncertainty that their light treatment was effective in terms of exposure and because they did not study sleep or sleep behaviour (Raap *et al.*, 2017b), it is still unknown how ALAN affects oxalate in birds. If oxalate is a cross-species biomarker of sleep debt then this could be used to quantify whether ALAN causes nestlings to suffer from sleep disruption. Moreover, oxalate would be a particularly useful tool when working with free-living small animals as they are often too small to be fitted with modern data loggers and behaviour is necessarily used as a proxy for sleep and sleep debt (Aulsebrook *et al.*, 2016). Data loggers are used to record brain activity which allows for a more precise and detailed measurement of sleep that cannot be obtained from behaviour. However, great tit nestlings are too small to be fitted with data loggers and sleep cannot be quantified behaviourally as they do not always exhibit the classical sleep position like adults do. Nonetheless, they are an important model system in evolutionary and environmental research, and are increasingly being used to study the effects of ALAN on behaviour and physiology (e.g. Da Silva *et al.*, 2014; Kempenaers *et al.*, 2010; Ouyang *et al.*, 2017; Raap *et al.*, 2017c; Sprau *et al.*, 2017; Sun *et al.*, 2017) and the impact of ALAN on nestling sleep would thus be of particular interest.

Oxalate is decreased in both sleep restricted rats and humans (Weljie *et al.*, 2015) and as ALAN affects nestling behaviour and physiology it is important to test whether it also affects oxalate in developing animals. Sleep likely plays a crucial role in metabolic processes. Weljie *et al.* (2015) investigated cross-species consequences via comprehensive metabolite profiling and showed that oxalate may provide a potential link between sleep loss and metabolic dysfunction and serve as a biomarker for sleep deprivation. Blood oxalate can decrease through several mechanisms as outlined in Weljie *et al.* (2015): reduced synthesis, increased gut microbiota processing and/or increased urinary clearance. Increases could occur through degradation of ascorbate, production by the liver and red blood cells or by increased dietary oxalate (Marengo and Romani, 2008). To examine whether ALAN affects oxalate in developing birds, we experimentally exposed wild great tit nestlings to two nights of artificial light (3 lux) and compared these to nestlings which were not exposed to ALAN.

## Methods

Plasma samples from a previous experiment were used in which free-living great tit nestlings were exposed to ALAN inside their nest box (see details in Raap *et al.*, 2016a, 2016b). In brief, our experiment was performed during the 2015 breeding season (between 8 and 25 May) in a resident nest box population of great tits in the surroundings of Wilrijk (Antwerp), Belgium (51°9′44″N, 4°24′15″E). This population was established in 1997 and has been monitored ever since (see e.g. Eens *et al.*, 1999; Van Duyse *et al.*, 2000, 2005; Janssens *et al.*, 2001; Rivera-Gutierrez *et al.*, 2010, 2012; Vermeulen *et al.*, 2016; Thys *et al.*, 2017).

**Figure 1:**
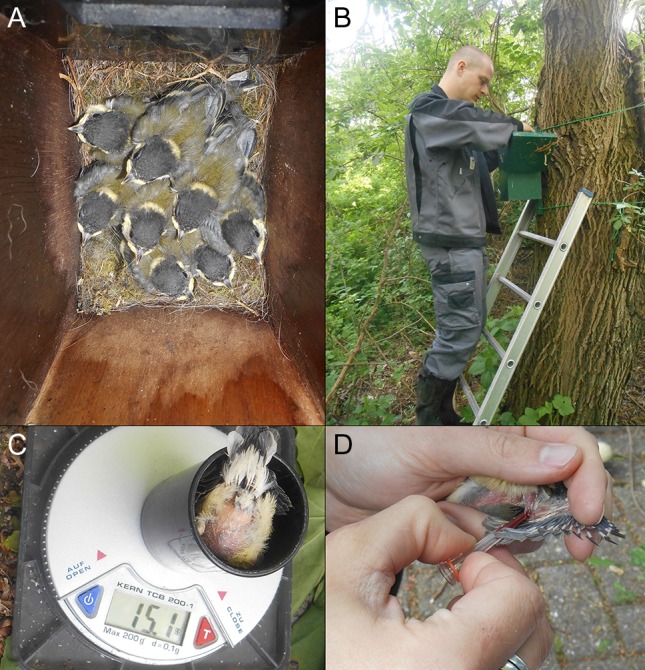
Fieldwork procedure. General fieldwork included taking great tit nestlings (**A**) out of their nest box (**B**) after which their body mass is taken (**C**) as well as a small blood sample (**D**).

The previous experiment included two groups of each 16 nests; a control and a light treated group (see details in Raap *et al.*, 2016a, 2016b). Nests with a similar hatching date and brood size (about seven) were used and otherwise randomly allocated to a treatment. In the light treated group, we obtained a blood sample (≤150 μl) from the brachial vein of all nestlings and subsequently weighed them (digital balance; Kern TCB 200-1) at baseline (Day 13 after hatching) and again after a two night exposure to ALAN (Fig. 1). Nestlings from the control ‘dark’ group were not exposed to ALAN, but were otherwise treated the same. Sampling was standardized for time and always occurred during the morning. The light treated group was exposed to 3 lux broad-spectrum white light as measured on the nest box bottom (ISO-Tech ILM-1335 light meter; Corby, UK). This intensity has been shown to disrupt sleep behaviour (Raap *et al.*, 2017c) and affect nestling physiology (Raap *et al.*, 2016a, 2016b).

We examined changes caused by ALAN on a within-individual level by taking two samples from each individual which we used to generate a single ‘change in oxalate’ measure (one metric per individual). This approach is especially important because there is high heterogeneity in physiological markers on the between-individual level within nests (see e.g. Vermeulen *et al.*, 2015; Casasole *et al.*, 2017). Moreover, effects of ALAN may differ greatly among individuals (see e.g. Raap *et al.*, 2016c). Our field-based experimental approach with free-living animals (contrary to laboratory studies), may offer useful insights about possible physiological effects of ALAN on developing animals.

Oxalate was quantified in 4 μl of plasma using the manufacturer’s instructions provided with the commercially available colorimetric assay (BioVision Inc., USA), similar to Ouyang *et al.* (2017) who also used great tits. Plasma samples had been stored at −80°C but had also been defrosted twice for previous analyses (see details in Raap *et al.*, 2016a, 2016b). In brief, we diluted samples with assay buffer to a total of 50 μl. In the same assay samples to generate a standard curve were included. Samples were subsequently incubated at 37°C for 1 h and absorbance was measured at 450 nm. We selected those samples of which sufficient plasma remained after previous analyses. Due to sample limitations we could not run duplicates although there was always some plasma left-over. All samples were within the assays limit. Samples of Days 13 and 15 from the same individual were kept on one plate, and two plates/assays were used in total. Overall we obtained repeated measurements (Days 13 and 15) of oxalate for 23 females and 21 males in the control group (14 nests) and 22 females and 22 males in the light group (13 nests). So, in total 88 great tit nestlings of known sex were sampled twice. Nestling sex was determined by molecular sexing (Griffiths *et al.*, 1998).

All statistical analyses were conducted in R 3.3.2 (R Core Team, 2016). We compared changes in oxalate from Day 13 to Day 15, between the control and light group. Changes in oxalate (expressed as a percentage from baseline, similar to Weljie *et al.*, 2015) were analysed by constructing a linear mixed model as data were normally distributed (lme4 package Bates *et al.*, 2015). The change in oxalate is unrelated to the level of oxalate on Day 13. Nest identity (NestID) was included as random factor to avoid pseudoreplication. The full model contained weight on Day 13 (covariate), sex (factor), treatment (factor) and the interaction between sex and treatment as explanatory variables. We included the interaction with sex as there may be sex-specific differences in physiology (Giordano *et al.*, 2015; Speakman *et al.*, 2015) and environmental sensitivity (reviewed in Jones *et al.*, 2009). *P*-values obtained by a stepwise backward model reduction (Zuur *et al.*, 2009) are given in the results and Tukey HSD tests were used for post-hoc analyses (lmerTest package Kuznetsova *et al.*, 2016).

## Results

**Figure 2:**
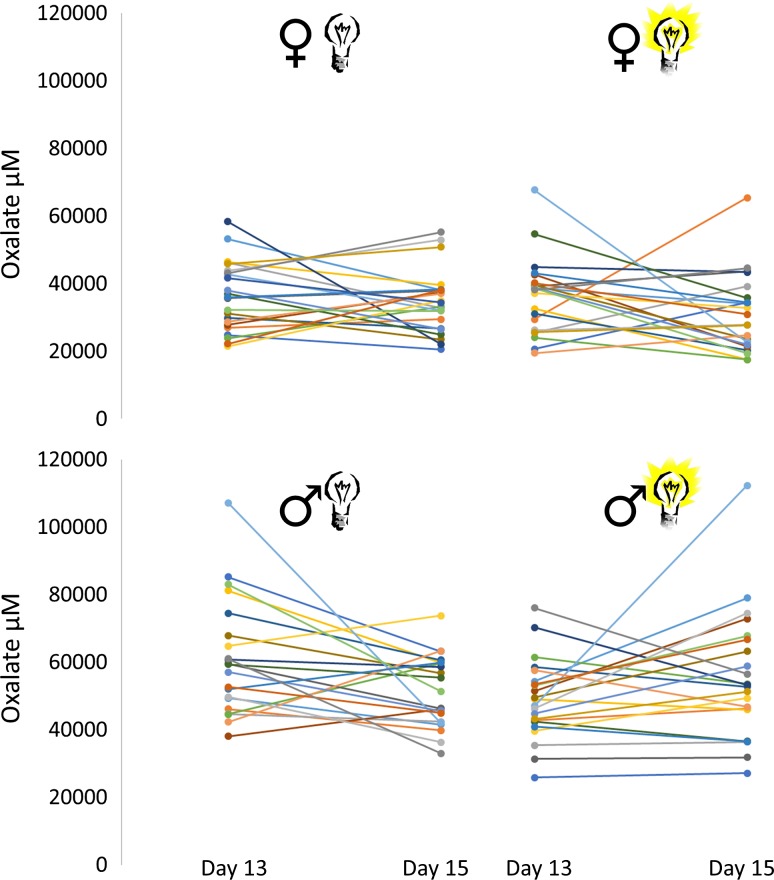
ALAN increased oxalate in male nestlings. Raw data of the change in oxalate after two nights is shown for females (top panels) and males (lower panels), in a natural dark situation (left panels) and for light exposed animals (right panels). Lines indicate unique individuals; sample sizes: ♀ dark = 23; ♂ dark = 21; ♀ light = 22; ♂ light = 22 nestlings. In males there was a significant effect of artificial light at night on oxalate levels (*t* = 2.01, *P* = 0.048; Table 3).

There was a sex-dependent effect of ALAN on oxalate (sex:treatment interaction: *F* = 4.994, *P* = 0.028; Tables 1 and 2). Light exposed males, but not females, showed increased levels of oxalate (≈15%; see Fig. 2 and Table 3). Male and female nestlings in a natural dark situation showed no change in oxalate from Day 13 to Day 15. Weight at Day 13 did not affect changes in oxalate (*F* = 0.050, *P* = 0.823).

**Table 1:** Statistical output of the full mixed effect model, effect of artificial light at night on oxalate.

	Estimate	SE	NumDF	DenDF	*F*-value	*P*-value
Intercept	−0.077	0.401				
Weight	0.006	0.026	1	83	0.050	0.823
Sex	−0.122	0.110	1	83	0.405	0.526
Treatment	−0.082	0.108	1	83	1.355	0.248
Sex:Treatment	0.343	0.154	1	83	4.978	**0.028**

A linear mixed model with ‘nest’ as random factor was used (lme4 package Bates *et al.*, 2015). In bold the significant *P*-value is indicated (*P*< 0.05), *N* = 88 individuals. *P*-values obtained after a stepwise backward regression can be found in Table 2. Estimates with their standard error (SE) are given. NumDF is nominator degrees of freedom, DenDF is denominator degrees of freedom.

**Table 2:** Statistical output of the model reduction, effect of artificial light at night on oxalate.

	NumDF	DenDF	*F*-value	elim.num	*P*-value
Weight	1	83	0.0503	1	0.8231
Sex	1	84	0.4542	kept	0.5022
Treatment	1	84	1.4112	kept	0.2382
Sex:Treatment	1	84	4.9941	kept	**0.0281**

A linear mixed model with ‘nest’ as random factor was used. Stepwise model reduction was performed (lmerTest package Kuznetsova *et al.*, 2016). In bold the significant *P*-value (*P* < 0.05) is indicated, *N* = 88 individuals. NumDF is nominator degrees of freedom, DenDF is denominator degrees of freedom, elim.num is the order in which a variable is removed from the model.

**Table 3:** Results of post-hoc analyses for the interaction between sex and treatment for the difference in oxalate between Day 13 and Day 15

	Estimate	SE	DF	*t*-Value	Lower CI	Upper CI	*P*-value
♀ dark	1.12	7.46	84	0.15	−13.72	15.97	0.8808
♂ dark	−10.80	7.81	84	−1.38	−26.33	4.74	0.1706
♀ light	−6.87	7.63	84	−0.9	−22.05	8.31	0.3705
♂ light	15.34	7.63	84	2.01	0.16	30.52	**0.0476**

Tukey HSD tests were used for post-hoc analyses (lmerTest package Kuznetsova *et al.*, 2016). Estimates give the difference between oxalate from Day 13 to Day 15 as a % from baseline (Day 13) and *P* values indicate whether this differs from 0. Sample sizes: ♀ dark = 23; ♂ dark = 21; ♀ light = 22; ♂ light = 22 nestlings. In bold the significant *P*-value (*P*< 0.05) is indicated. Standard error (SE) and confidence interval (CI) of the estimates are given. See Fig. 2 for the raw data of the change in oxalate.

## Discussion

We found a sex-dependent effect of ALAN on oxalate, a potential biomarker for sleep debt (Weljie *et al.*, 2015). The effect of ALAN on oxalate only manifested itself in male nestlings and in the opposite direction as we had expected, an increase instead of a decrease. In the following, we discuss these sex-dependent effects of ALAN on oxalate and the potential of oxalate as a proxy for sleep debt in developing birds.

### Sex-dependent effect of ALAN on oxalate

Males and females differ in their behaviour, physiology and their response to the environment which may explain that effects on oxalate were only found in males. Oxalate could increase through degradation of ascorbate, production by the liver and red blood cells or by increased dietary oxalate (Marengo and Romani, 2008) and there are several indications why oxalate could be affected in a sex-dependent manner. First, sleep behaviour (including its duration) is strongly sex-dependent in great (Stuber *et al.*, 2015a, 2017) and blue tits (Steinmeyer *et al.*, 2010), and adult male and female great tits use different sleep strategies depending on their metabolic requirements (Stuber *et al.*, 2015b). Contrary to females, males decrease their sleep duration with an increased basal metabolic rate. Second, male and female great tit nestlings may also differ in their physiology besides possible behavioural differences. For example, male nestlings have higher nitric oxide levels (Raap *et al.*, 2017a), and nutritional conditions during development had a sex-dependent effect on the oxidative status of great tit nestlings, with females being more sensitive to nutritional stress indicating sex-specific allocation priorities (Giordano *et al.*, 2015). Third, the response towards ALAN is potentially sex-dependent as adult females showed a slightly stronger reduction than males in sleep amount (% of sleep; amount divided by the total time spent inside the nest box) when subjected to ALAN in February (Raap *et al.*, 2015). However, whether this is true during the breeding season and for developing nestlings remains to be tested. Generally speaking males are often more susceptible to environmental conditions than females although effects are small (reviewed by Jones *et al.*, 2009). Overall, this implies that male and female nestlings may differ in their sleep behaviour, physiology and response to the environment, which could contribute to the observed differences in oxalate. Finally, we need to consider that as this is the first study in developing animals in the wild, our results may also be false positive. The effect is not particularly strong and in the direct opposite of predictions. However, we used a repeated measures design to look at changes within the same individuals, which increases statistical power (Seltman, 2013) and gives confidence in the obtained results. Nonetheless, further experimental and comparative work is needed to determine and validate our results.

Increased begging might have led to increased food provisioning by the parents and thus higher oxalate levels in males. When nestlings are exposed to ALAN they start begging for food which they never do during a dark night. Although this needs to be examined it could trigger females (and to an extent indirectly also males) to feed their nestlings more, especially during the morning. Our samples were obtained during the morning between 08:00 and 12:00. During this period nestlings could have received a substantial amount of food and because male nestlings are larger than females (average of Day 13–15, respectively, 15.9 ± 0.230 and 15.2 ± 0.231 g; Raap *et al.*, 2016a) they might have received more food (e.g. Anderson *et al.*, 1993) and thus more dietary oxalate. However, whether male nestlings did effectively obtain more food, that also contains oxalate, needs to be determined. Doing so will be challenging because, besides other practical issues, nestling sex can only be determined genetically. Another potentially contributing mechanism could be through the disruption of the gut microbiota by ALAN. This could have led to reduced processing and thus accumulation of oxalate in light exposed nestlings but this requires further investigation and does not explain the sex-dependent effect. To conclude, the increase of oxalate by ALAN may therefore be unrelated to any changes in sleep.

### Oxalate as a proxy for sleep debt in developing birds

Because of the inability to directly measure sleep amount or disruption in nestlings we cannot be entirely certain about the relationship between sleep loss and oxalate levels in developing nestlings. While nestlings showed more begging at night when exposed to ALAN (Raap *et al.*, 2016c), we cannot be sure that they spent more time awake. Awakenings at night are a normal part of the adult tits’ sleep patterns, however, ALAN clearly disrupts adult sleep behaviour (Raap *et al.*, 2015, 2016c). It therefore seems very likely that great tit nestlings disturbed by ALAN (Raap *et al.*, 2016c) also effectively slept less, but whether an increase in oxalate is related to sleep loss remains to be tested. Furthermore, whether oxalate can be used as a reliable biomarker of sleep loss in (developing) birds and whether it is direct or indirectly affected by ALAN will require further research.

Effects of sleep loss on oxalate in developing animals may differ from those in adults, where in rats and humans a decrease was found. Sleep differs between developing and adult animals in both mammals and birds (reviewed in Rattenborg and Martinez-Gonzalez, 2015), with for example the amount of rapid eye movement (REM) sleep being higher in developing animals than in adults. Following sleep deprivation a rebound mainly occurs in REM sleep. Sleep loss may thus be different for developing animals compared to adults. This difference may subsequently be reflected in differences in how oxalate is affected. Ouyang *et al.* (2017) examined effects of light pollution on oxalate levels in adult great tits. They claimed that great tits exposed to white light had higher nightly activity and linked this to a decrease in oxalate from March to May. However, there are several serious issues with these results (Raap *et al.*, 2017b), warranting further study. For example, there is high uncertainty that their light treatment was effective in terms of exposure and the claims about a relationship between sleep loss (which was not measured) and physiological effects seems to be premature. Furthermore, Weljie *et al.* (2015) showed that in rats (but not in humans) the reduction of oxalate was related to increased oxidative stress due to sleep deprivation. We would thus expect that sleep deprivation in developing great tits might be associated with oxidative status as well as reduction in oxalate. However, we previously showed that our treatment of two nights ALAN did not affect the oxidative status of developing great tits (Raap *et al.*, 2016a).

Our experimental treatment may not have restricted sleep sufficiently to be detected by a decrease in oxalate. The laboratory study by Weljie *et al.* (2015) revealed that oxalic acid is depleted after sleep restriction in male rats over a period of 5 days, while allowing them to sleep for only 4 h per day. In our study nestlings were exposed to ALAN continuously during two nights and the amount of sleep loss each night might be more severe than the sleep reduction in the study by Weljie *et al.* (2015) although their treatment was three days longer. Therefore, the reduction in sleep by ALAN over two nights could be insufficient to be detected using oxalate, although it affected body mass gain, haptoglobin and nitric oxide (Raap *et al.*, 2016a, 2016b). Future studies may, however, build upon our results and use longer periods of sleep restriction.

### Conclusions

We have shown here for the first time that a brief exposure of only two nights to ALAN affects oxalate levels in developing wild animals. This provides further evidence of the physiological effects of ALAN during early-life, such as increased haptoglobin and decreased nitric oxide (Raap *et al.*, 2016b), in addition to the behavioural effects, such as increased begging behaviour and likely disrupted sleep (Raap *et al.*, 2016c). The sex-dependent effect on oxalate might indicate different physiological coping mechanisms by developing great tits, which requires further investigation. Experiments using humans and rats showed that oxalate is a potential biomarker of sleep loss (Weljie *et al.*, 2015), and while its use in free-living birds and developing animals is promising it requires further investigation. The increasing illumination of the night is a serious threat as it disrupts circadian rhythms, physiology and behaviour. Early-life exposure could have long-lasting effects throughout adulthood, reducing survival and reproduction. With the progressive, worldwide increase of light pollution, the physiological effects of this stressor, especially during development, should receive greater attention. Urbanization is not only associated with light pollution but with many other anthropogenic stressors such as noise pollution, and new methods and approaches are necessary to understand the consequences of increasing human pressure on free-living animals.

## Data accessibility

The dataset supporting this article was uploaded to the Zenodo digital repository doi: 10.5281/zenodo.831995.
